# Cholesterol Assimilation by *Lactobacillus* Probiotic Bacteria: An *In Vitro* Investigation

**DOI:** 10.1155/2014/380316

**Published:** 2014-09-11

**Authors:** Catherine Tomaro-Duchesneau, Mitchell L. Jones, Divya Shah, Poonam Jain, Shyamali Saha, Satya Prakash

**Affiliations:** ^1^Biomedical Technology and Cell Therapy Research Laboratory, Departments of Biomedical Engineering, Physiology, and Artificial Cells and Organs Research Center, Faculty of Medicine, McGill University, 3775 University Street, Montreal, QC, Canada H3A 2B4; ^2^Micropharma Limited, 4200 Saint Laurent Boulevard, Unit 409, Montreal, QC, Canada H2W 2R2; ^3^Faculty of Dentistry, McGill University, Montreal, QC, Canada H3A 0C7

## Abstract

Excess cholesterol is associated with cardiovascular diseases (CVD), an important cause of mortality worldwide. Current CVD therapeutic measures, lifestyle and dietary interventions, and pharmaceutical agents for regulating cholesterol levels are inadequate. Probiotic bacteria have demonstrated potential to lower cholesterol levels by different mechanisms, including bile salt hydrolase activity, production of compounds that inhibit enzymes such as 3-hydroxy-3-methylglutaryl coenzyme A, and cholesterol assimilation. This work investigates 11 *Lactobacillus* strains for cholesterol assimilation. Probiotic strains for investigation were selected from the literature: *Lactobacillus reuteri* NCIMB 11951, *L. reuteri* NCIMB 701359, *L. reuteri* NCIMB 702655, *L. reuteri* NCIMB 701089, *L. reuteri* NCIMB 702656, *Lactobacillus fermentum* NCIMB 5221, *L. fermentum* NCIMB 8829, *L. fermentum* NCIMB 2797, *Lactobacillus rhamnosus* ATCC 53103 GG, *Lactobacillus acidophilus* ATCC 314, and *Lactobacillus plantarum* ATCC 14917. Cholesterol assimilation was investigated in culture media and under simulated intestinal conditions. The best cholesterol assimilator was *L. plantarum* ATCC 14917 (15.18 ± 0.55 mg/10^10^ cfu) in MRS broth. *L. reuteri* NCIMB 701089 assimilated over 67% (2254.70 ± 63.33 mg/10^10^ cfu) of cholesterol, the most of all the strains, under intestinal conditions. This work demonstrates that probiotic bacteria can assimilate cholesterol under intestinal conditions, with *L. reuteri* NCIMB 701089 showing great potential as a CVD therapeutic.

## 1. Introduction

Early studies by Anitschkow demonstrated that cholesterol administration results in symptoms of atherosclerosis [1], contributing to the lipid hypothesis, formulated by Duff and McMillan, which proposed an association between cholesterol and cardiovascular diseases (CVD) [2]. CVD are the leading cause of global mortality and morbidity and kill an estimated 16.7 million people worldwide [3]. Coronary artery disease (CAD), the most common CVD, is the leading cause of death and accounts for 7.25 million deaths globally [4]. The first line of treatment for CAD, dietary and lifestyle interventions, has proven inadequate. Pharmacological agents are being administered to target elevated low-density lipoprotein (LDL) levels [5, 6], including 3-hydroxy-3-methylglutaryl coenzyme A (HMG-CoA) reductase inhibitors (statins), fibric acids, high-density lipoprotein stimulators (nicotinic acids), cholesterol absorption inhibitors (ezetimibe), and bile acid sequestrants. These pharmaceutics, however, have important limitations, with only 38% of dyslipidemia and 18% of CAD patients attaining the National Cholesterol Education Program goals [7]. Statins, the fundamental therapy for reducing LDL levels [8], fail to allow the majority of patients to meet their lipid goals [7, 9, 10]. There is a dire need for additional therapeutic modalities to lower cholesterol levels.

There has been increasing interest in probiotics, “microorganisms which when administered in adequate amounts confer a health benefit on the host,” research for the development of biotherapeutics [11, 12]. In recent years, attention has been given to the ability of probiotic cells to reduce lipids and cholesterol levels [13], with several proposed mechanisms of action. One mechanism, bile salt hydrolase activity, is described in a recent review [14]. In addition, bacteria have been reported to assimilate cholesterol [15, 16], thereby lowering luminal cholesterol levels available for absorption. Moreover,* Lactobacillus* bacteria can produce ferulic acid (FA) [17, 18], which can inhibit hepatic HMG-CoA reductase and promote the excretion of acidic sterol [19]. With the demonstrated cholesterol-lowering properties of probiotic bacteria, further research is required to investigate the mechanism(s) by which the bacteria decrease cholesterol levels and to select bacteria capable of exerting cholesterol-lowering effects.

The goal of the presented work is to investigate* Lactobacillus *strains for their potential to assimilate cholesterol in both bacterial media and under simulated gastrointestinal conditions. This work provides grounds for future investigations to select a probiotic bacterial strain as a cholesterol-lowering therapeutic.

## 2. Materials and Methods

### 2.1. Bacterial Growth Media and Chemicals

De Man-Rogosa-Sharpe (MRS) broth was purchased from Fisher Scientific and prepared according to the manufacturer's instructions. Cholesterol-polyethylene glycol (PEG) 600 was purchased from Sigma-Aldrich (Oakville, ON, Canada). Water was purified with an EASYpure Reverse Osmosis System and a NANOpure Diamond Life Science (UV/UF) ultrapure water system from Barnstead Scientific Instrumentation (Dubuque, IA, USA). All other chemicals were of analytical or HPLC grade and purchased from commercial sources.

### 2.2. Bacterial Strains and Culture Conditions

The bacterial strains used in this study are listed in Table 1.* Lactobacillus fermentum* NCIMB 5221,* Lactobacillus fermentum* NCIMB 2797,* Lactobacillus fermentum* NCIMB 8829,* Lactobacillus reuteri* NCIMB 701359,* Lactobacillus reuteri* NCIMB 11951,* Lactobacillus reuteri* NCIMB 701089,* Lactobacillus reuteri* NCIMB 702656, and* Lactobacillus reuteri* NCIMB 702655 were purchased from the National Collection of Industrial, Food and Marine Bacteria (NCIMB, Aberdeen, Scotland, UK).* Lactobacillus rhamnosus* ATCC 53103 GG,* Lactobacillus acidophilus* ATCC 314, and* Lactobacillus plantarum* ATCC 14917 were purchased from Cedarlane Labs (Burlington, ON, Canada). All strains were kept as frozen stocks and stored at −80°C in MRS broth containing 20% (v/v) glycerol. Prior to any assay, a MRS-agar plate was streaked from the frozen stock to ensure purity and incubated at 37°C with 5% CO_2_ for 24 h. One colony from the agar plate was used to inoculate 10 mL MRS broth which was then incubated at 37°C for 24 h, prior to any experimental assay. Bacterial cell viabilities were determined using standard colony counting methods. Briefly, 10-fold serial dilutions were prepared using 0.85% (w/v) NaCl. Diluted bacterial samples were streaked on MRS-agar plates which were then incubated at 37°C and 5% CO_2_ for 48 h. Colonies were counted from each plate and the colony forming units (cfu) were recorded. All viability tests were performed in triplicate to ensure accuracy and reproducibility.

### 2.3. Determining Probiotic Cholesterol Assimilation in MRS

The probiotic* Lactobacillus *strains were investigated for their capability to assimilate cholesterol in MRS broth. Cholesterol-PEG 600 was added to MRS broth at a final concentration of 100 *μ*g/mL. A 1% (v/v) inoculum of each overnight probiotic culture was added to MRS-cholesterol-PEG 600 and incubated at 37°C for 24 h. Following incubation, viability was measured by standard colony counting methods. For cholesterol analysis, the probiotic suspensions were centrifuged at 4000 rpm for 10 min at 4°C using a Napco 2028R centrifuge (Fisher Scientific, Ottawa, ON, Canada) and the supernatants containing nonassimilated cholesterol were collected.

Cholesterol concentrations in the different suspensions were determined using a protocol modified from Rudel and Morris [30]. Briefly, 500 *μ*L of 33% (w/v) KOH and 1 mL absolute ethanol were added to 500 *μ*L of the samples. The solutions were then vortexed for 1 min and incubated at 37°C for 15 min followed by cooling to room temperature. For phase separation, 1 mL of deionized water and 1.5 mL of hexanes were added to the solutions and vortexed for 1 min. The phases were then allowed to separate at room temperature. Subsequently, 500 *μ*L of the hexane layer was transferred into a glass tube and the solvent was evaporated under a flow of nitrogen gas. Once dried, 1 mL of 50 mg/dL o-phthalaldehyde reagent prepared in acetic acid was added and the samples were mixed. Following mixing, 250 *μ*L of concentrated H_2_SO_4_ was added to each tube and the solutions were vortexed for 1 min, followed by incubation for 20 min at room temperature. The resulting absorbance was read at 570 nm using a UV spectrophotometer Victor3 V 1420 Multilabel Counter (Perkin Elmer, Boston, MA, USA). A standard curve of absorbance versus cholesterol concentrations was generated using the cholesterol concentrations: 0, 3.91, 7.81, 15.63, 31.25, 62.5, 125, 250, and 500 *μ*g/mL cholesterol in MRS (*R* ^2^ = 0.9875).

The cholesterol assimilated by probiotic* Lactobacillus* strains was determined as follows:

(1) cholesterol  assimilated  (μg/mL) =[cholesterol  (μg/mL)]0 h−[cholesterol  (μg/mL)]24 h.

Cholesterol assimilated by each* Lactobacillus *strain was also calculated in terms of percent cholesterol assimilation:

(2) %  cholesterol  assimilated =[cholesterol  assimilated  (μg/mL)cholesterol  (μg/mL)0 h]×100%.

Cholesterol assimilated by each* Lactobacillus *strain was calculated considering a dose of 10^10^ cells:

(3) cholesterol  assimilated  (mg/mL)probiotic  cell  viability  (cfu/mL)×1010.

Samples and standards were tested in triplicate to ensure accuracy and reproducibility.

### 2.4. Determining Probiotic Cholesterol Assimilation under Simulated Intestinal Conditions

The* Lactobacillus *strains were investigated for their capability to assimilate cholesterol under simulated intestinal conditions. Simulated intestinal fluid was prepared according to US Pharmacopeia, with modifications [31]. Briefly, simulated intestinal fluid consisted of 0.85% (w/v) NaCl, 6.8 g/L potassium phosphate monobasic, 1.5 g/L Oxgall, 3.5 g/L glucose, and 10 g/L pancreatin. The pH was adjusted to 6.8 by the addition of 2 M NaOH.

Cholesterol-PEG 600 was added to the simulated intestinal fluid at a final concentration of 100 *μ*g/mL. A 1% (v/v) inoculum of each overnight probiotic culture was added to the simulated intestinal fluid. The tubes were then incubated at 37°C for 24 h on a rotary shaker set at 100 rpm. Following a 24-hour incubation, viability was determined by standard colony counting methods. For cholesterol analysis, the probiotic suspensions were centrifuged at 4000 rpm for 10 min at 4°C to collect the supernatant. Cholesterol assimilation was determined, as previously described. A standard curve of absorbance versus cholesterol concentrations in simulated intestinal fluid was generated using the concentrations: 0, 3.91, 7.81, 15.63, 31.25, 62.5, 125, 250, and 500 *μ*g/mL cholesterol (*R* ^2^ = 0.9875). Samples and standards were tested in triplicate to ensure accuracy and reproducibility.

### 2.5. Statistical Analysis

Experimental results are expressed as means ± standard error of the mean (SEM). Statistical analysis was carried out using SPSS Version 17.0 (Statistical Product and Service Solutions, IBM Corporation, New York, NY, USA). Linear regression was performed for generating standard curves. Statistical comparisons were carried out using the general linear model, followed by multiple comparisons of the means using Tukey's post hoc analysis. Statistical significance was set at *P* < 0.05 and* P* values less than 0.01 were considered highly significant.

## 3. Results

### 3.1. Cholesterol Assimilation in MRS

**Figure 1:**
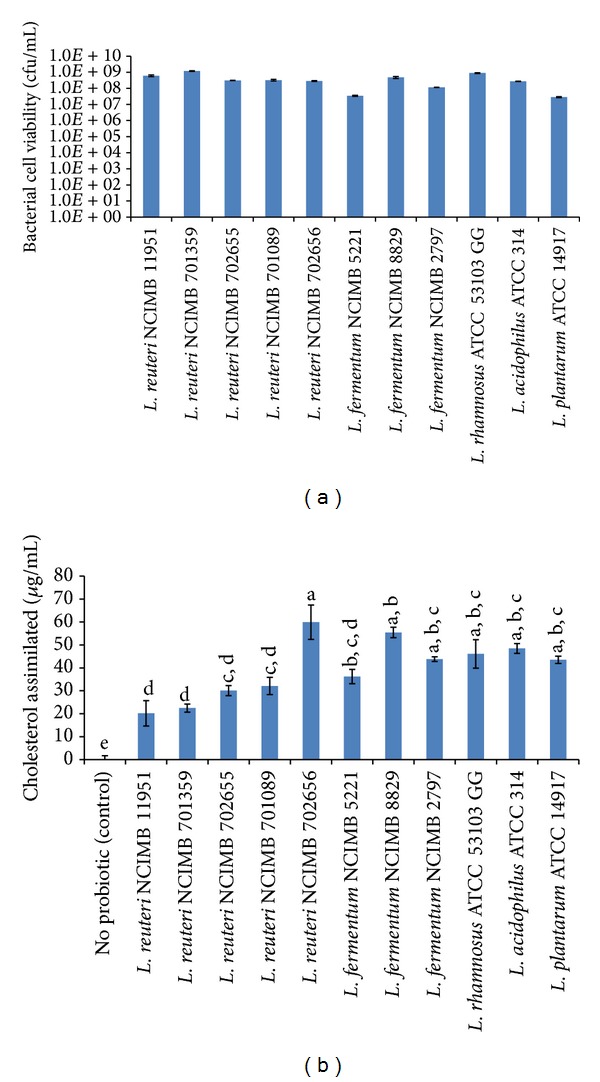
(a) Viability and (b) cholesterol assimilation of probiotic* Lactobacillus* in MRS containing 100 *μ*g/mL of cholesterol, following 24 h of incubation. Data is represented as means ± SEM, *n* = 3. Tukey's homogeneous subsets generated from pairwise comparisons are represented as a, b, c, d, and e, with “a” representing the most significant subset from control.

The capability of 11 probiotic* Lactobacillus *strains to assimilate cholesterol in MRS media was determined. The viability (expressed as cfu/mL) of the probiotic cells was investigated upon incubation with 100 *µ*g/mL water-soluble cholesterol-PEG 600 in MRS. All probiotic cells under investigation were viable after incubation with cholesterol in the growth media for 24 h at 37°C, as shown in Figure 1(a). The cell viability ranged from 2.87 ± 0.176 × 10^7^ cfu/mL for* L. plantarum *ATCC 14917 to 1.18 ± 0.0504 × 10^9^ cfu/mL for* L. reuteri *NCIMB 701359. All the strains investigated were successful (*P* < 0.001) at assimilating cholesterol following 24 h of incubation in cholesterol-containing MRS, as seen in Figure 1(b). The control sample, containing no probiotic, demonstrated no cholesterol assimilation, as expected. Six* Lactobacillus *strains (subset “a” determined by Tukey's pairwise comparison) were shown to be significantly the best (*P* < 0.05) at assimilating cholesterol in MRS:* L. reuteri *NCIMB 702656 (59.94 ± 7.49 *μ*g/mL),* L. fermentum *NCIMB 8829 (55.44 ± 2.29 *μ*g/mL),* L. acidophilus *ATCC 314 (48.45 ± 2.13 *μ*g/mL),* L. rhamnosus *GG ATCC 53103 (46.09 ± 6.19 *μ*g/mL),* L. fermentum *NCIMB 2797 (43.79 ± 1.04 *μ*g/mL), and* L. plantarum *ATCC 14917 (43.52 ± 1.59 *μ*g/mL). The strains with the least cholesterol assimilation (subset “d”) were* L. reuteri *NCIMB 11951 (20.18 ± 5.55 *μ*g/mL),* L. reuteri *NCIMB 701359 (22.49 ± 1.76 *μ*g/mL),* L. reuteri *NCIMB 702655 (30.07 ± 2.23 *μ*g/mL),* L. reuteri *NCIMB 701089 (32.10 ± 3.75 *μ*g/mL), and* L. fermentum *NCIMB 5221 (36.21 ± 3.1 *μ*g/mL).

The amount of cholesterol assimilated by the probiotic* Lactobacillus *strains in terms of a dose of 10^10^ cells was calculated. The results indicate that all of the strains significantly (*P* < 0.001) assimilated cholesterol, in terms of mg cholesterol assimilated per 10^10^ cells in MRS, as shown in Table 2. The results obtained, when bacterial cell counts were taken into account, were different from those previously described. Indeed, when normalized for viability counts, one probiotic strain,* L. plantarum *ATCC 14917, assimilated the most cholesterol, with 15.18 ± 0.55 mg of cholesterol assimilated per 10^10^ cells (*P* < 0.05, subset “a”). The* Lactobacillus *strains that assimilated the least cholesterol, in terms of assimilation by 10^10^ cfu (subset “e”), were* L. reuteri *NCIMB 11951 (0.33 ± 0.09 mg/10^10^ cfu),* L. reuteri *NCIMB 701359 (0.19 ± 0.02 mg/10^10^ cfu),* L. reuteri *NCIMB 702655 (0.96 ± 0.07 mg/10^10^ cfu),* L. reuteri *NCIMB 701089 (0.99 ± 0.12 mg/10^10^ cfu),* L. fermentum *NCIMB 8829 (1.16 ± 0.05 mg/10^10^ cfu),* L. rhamnosus *ATCC 53103 GG (0.52 ± 0.07 mg/10^10^ cfu), and* L. acidophilus *ATCC 314 (1.79 ± 0.08 mg/10^10^ cfu).

### 3.2. Cholesterol Assimilation under Simulated Intestinal Conditions

**Figure 2:**
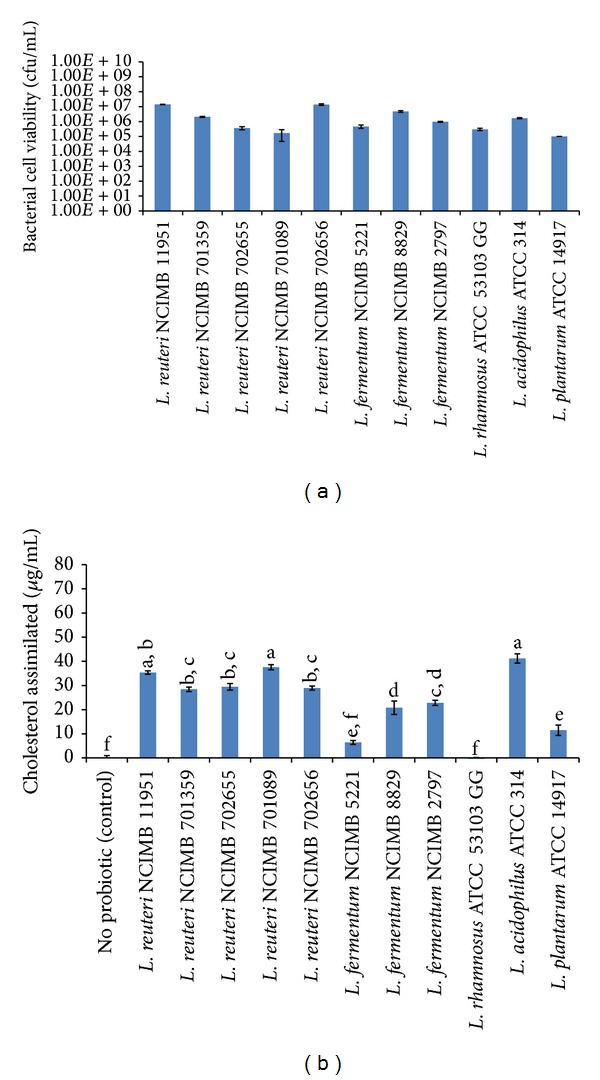
(a) Viability and (b) cholesterol assimilation of probiotic* Lactobacillus* in simulated intestinal fluid containing 100 *μ*g/mL of cholesterol, following 24** **h of incubation. Data is represented as means ± SEM, *n* = 3. Tukey's homogeneous subsets generated from pairwise comparisons are represented as a, b, c, d, e, and f, with “a” representing the most significant subset from control.

The* Lactobacillus *strains were further investigated for their capability to assimilate cholesterol under simulated intestinal conditions. The viability of the probiotic* Lactobacillus *strains was investigated upon incubation with 100 *μ*g/mL water-soluble cholesterol-PEG 600 in simulated intestinal fluid. Following 24 h of incubation at 37°C, all the probiotic cells under investigation were found to be viable, as shown in Figure 2(a). Specifically, the bacterial cell viability ranged from 1 × 10^5^ ± 0.01 × 10^5^ cfu/mL for* L. plantarum *ATCC 14917 to 1.41 × 10^7^ ± 0.046 × 10^7^ cfu/mL for* L. reuteri *NCIMB 11951. In terms of cholesterol assimilation, the control, containing no probiotic, demonstrated no cholesterol assimilation, as expected. All the strains of* Lactobacillus* under investigation, except* L. rhamnosus *ATCC 53103 GG (−0.43 ± 0.61 *μ*g/mL), were successful (*P *< 0.001) at assimilating cholesterol (expressed as *μ*g/mL), as demonstrated in Figure 2(b). Three* Lactobacillus *strains (subset “a”) were shown to be significantly the best (*P *< 0.05) at assimilating cholesterol under simulated intestinal conditions:* L. reuteri *NCIMB 11951 (35.36 ± 0.72 *μ*g/mL),* L. reuteri *NCIMB 701089 (37.58 ± 1.06 *μ*g/mL), and* L. acidophilus *ATCC 314 (41.20 ± 1.92 *μ*g/mL).

Similar to the studies in MRS broth, the amount of cholesterol assimilated by the probiotic* Lactobacillus *strains in terms of a dose of 10^10^ cells following 24 h of incubation under simulated conditions was calculated, as shown in Table 3. Indeed, when bacterial cell counts of each strain are accounted for, the best strains are different from those previously described. However, the results indicate that all of the strains significantly (*P* < 0.05) assimilated cholesterol, in terms of mg cholesterol assimilated per 10^10^ cells under simulated intestinal conditions, except for* L. rhamnosus *ATCC 53103 GG (−14.19 ± 20.39 mg of cholesterol assimilated per 10^10^ cells). One probiotic strain,* L. reuteri *NCIMB 701089 (*P* < 0.05, subset “a”), demonstrated the most assimilation, when cholesterol assimilation is normalized for viability counts, with 2254.70 ± 63.33 mg of cholesterol assimilated per 10^10^ cells.

## 4. Discussion

**Figure 3:**
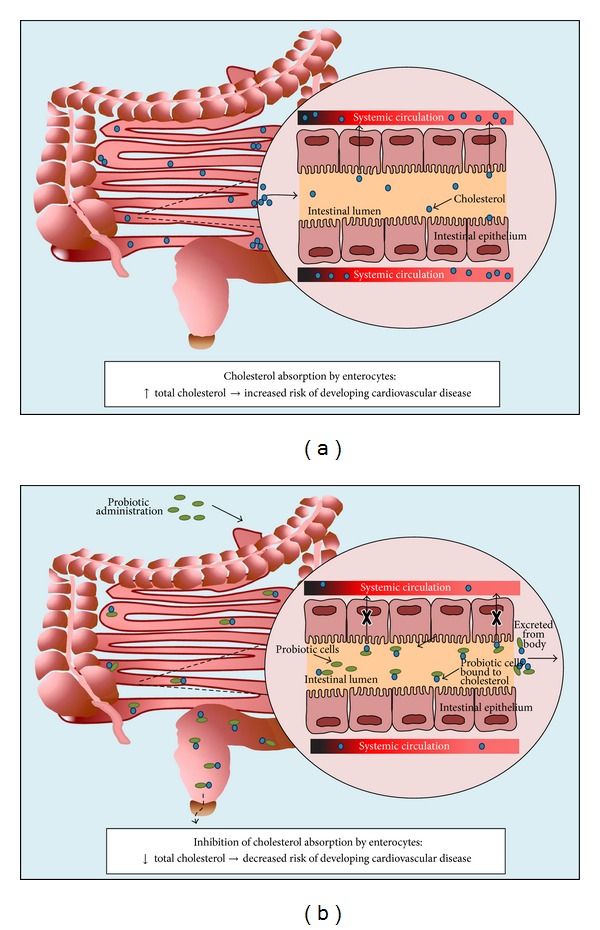
Schematic representation of probiotic cholesterol assimilation mechanism. (a) Cholesterol absorption by the intestinal enterocytes increases cardiovascular disease risks. (b) Probiotic administration enhances cholesterol assimilation, leading to the excretion of nonmetabolized cholesterol and other lipid molecules decreasing cardiovascular disease risks.

The risk of developing CAD, the leading cause of death, is directly associated with elevated cholesterol levels. With the increasing prevalence of CAD and the lack of a successful therapeutic, there is an important need for a novel therapeutic approach. Recent work on the gut microbiome has led to investigations of probiotic formulations for health disorders, including metabolic syndrome, inflammatory bowel disease, and allergies [11, 21, 32, 33]. Probiotic bacteria are advantageous as they are naturally found in foods such as yoghurt, are inexpensive, and are generally regarded as safe (GRAS). Of interest are the recent results demonstrating that probiotic bacteria have significant cholesterol-lowering properties [14, 21]. The hypocholesterolemic effects of probiotic bacteria have been linked to intrinsic bile salt hydrolase activity [14], cholesterol assimilation and incorporation in cellular membranes [15, 16], and the production of compounds, such as FA [17, 18], that can inhibit the activity of enzymes, including HMG-CoA reductase [19]. Cholesterol assimilation by probiotic bacteria in the gastrointestinal tract would allow for the reduction of cholesterol absorption by enterocytes and excretion of the cholesterol from the host, as depicted in Figure 3. This would, in turn, lead to a decreased risk of developing CAD. The goal of the presented work was to investigate probiotic strains for their ability to assimilate cholesterol from bacterial culture media, as well as under simulated intestinal conditions.

Previous groups have demonstrated that certain probiotic bacterial strains can assimilate cholesterol [34, 35]. Screening for cholesterol-lowering properties,* in vitro*, has become an important criterion in the selection of bacterial strains for* in vivo *probiotic investigations. We investigated* Lactobacillus *strains, selected from previous studies, for their ability to assimilate cholesterol. Initially, MRS bacterial culture media was supplemented with cholesterol and the bacterial strains were added for 24 h of incubation. All the bacterial strains were shown to successfully assimilate cholesterol but with high variability across the species and strains. There were six* Lactobacillus *strains that assimilated the most cholesterol in MRS broth:* L. reuteri *NCIMB 702656,* L. fermentum *NCIMB 8829,* L. acidophilus *ATCC 314,* L. rhamnosus *GG ATCC 53103,* L. fermentum *NCIMB 2797, and* L. plantarum *ATCC 14917. Cholesterol assimilation was as high as 59.94 ± 7.49 *μ*g/mL, for* L. reuteri *NCIMB 702656. Studies by previous groups have demonstrated cholesterol assimilation in the same range, with Bordoni et al. demonstrating that* Bifidobacterium longum *subspecies* infantis *ATCC assimilates 40 *μ*g/mL and* Bifidobacterium bifidum *MB 109 assimilated 50 *μ*g/mL of cholesterol in MRS broth [36]. Similarly, Yu et al. demonstrated that probiotic strains could assimilate cholesterol in the range of 14–22 *μ*g/mL [37].

As the previous experiments used bacterial growth media, we further investigated probiotic cholesterol assimilation under simulated intestinal conditions, to more closely mimic* in vivo *conditions, a first in the literature. The results, as in MRS, demonstrated a high variability of cholesterol assimilation over the various bacterial strains and species. Under these conditions, the* Lactobacillus *strains that assimilated the most cholesterol were* L. reuteri *NCIMB 11951,* L. reuteri *NCIMB 701089, and* L. acidophilus *ATCC 314, with cholesterol assimilation as high as 41.20 ± 1.92 *μ*g/mL, for* L. acidophilus *ATCC 314.

We also investigated how much cholesterol would be assimilated based on 10^10^ bacterial cells, representative of a typical probiotic dose. When cell counts of each strain were accounted for, under simulated intestinal conditions,* L. reuteri *NCIMB 701089 was the best (*P* < 0.05) assimilator with 2254.70 ± 63.33 mg of cholesterol assimilated per 10^10^ cells. Hypercholesterolemia is defined as having a serum cholesterol level over 240 mg/dL [38]. With this number in mind, we hypothesize that the administration of the probiotic strains, especially* L. reuteri *NCIMB 701089, could lower cholesterol levels significantly, although animal studies are required to evaluate its efficacy. One concern is the fact that recent work, by Madani et al., questions the use of* in vitro *cholesterol reducing activity assays as predictors of* in vivo *cholesterol-lowering activity [39]. With this in mind, it is clear that there is a need for additional work into strains, such as* L. reuteri *NCIMB 701089, prior to its use as a cholesterol-lowering therapeutic. Future work may focus on investigations into other cholesterol-lowering properties, including screening for bile salt hydrolase activity. In terms of cholesterol assimilation, the specific mechanism by which the cholesterol is removed from the supernatant should be determined. Ideally, a probiotic that would influence multiple targets, using bile salt hydrolase activity, reducing HMG-CoA reductase activity, and assimilating cholesterol, would be developed. In addition, a probiotic formulation could be developed as a combination therapy with pharmaceutics such as statins.

## 5. Conclusion

These results provide an initial screening of probiotic strains for their efficacy as cholesterol-lowering therapeutics via cholesterol assimilation. The capability of probiotic* Lactobacillus *strains to remove cholesterol from media, especially under simulated intestinal conditions, demonstrates their potential use as cholesterol-lowering agents. Moreover, the data suggests that* L. reuteri *NCIMB 701089 should be further characterized for its capability to lower cholesterol, using both* in vitro *and* in vivo *investigations. This work is an initial step for the development of a successful cholesterol-lowering probiotic therapeutic.

## Figures and Tables

**Table 1:** Probiotic bacteria selected for investigations into cholesterol assimilation based on previous cholesterol-lowering work.

Bacterial species	Strain	Reference(s)
*Lactobacillus reuteri *	NCIMB 11951	[14, 20]
	NCIMB 701359	
	NCIMB 702655	
	NCIMB 701089	
	NCIMB 702656	

*Lactobacillus fermentum *	NCIMB 5221	[21]
	NCIMB 8829	
	NCIMB 2797	

*Lactobacillus rhamnosus *	GG ATCC 53103	[22]

*Lactobacillus acidophilus *	ATCC 314	[23–25]

*Lactobacillus plantarum *	ATCC 14917	[26–29]

**Table 2:** Percent cholesterol assimilation by *Lactobacillus *strains in MRS containing 100 *μ*g/mL of cholesterol-PEG 600 for 24 h and the amount of cholesterol assimilation expected in a probiotic dose containing 10^10^ cells.

Probiotic strain	Cholesterol assimilated (%)	Cholesterol assimilated (mg/10^10^ cfu)
Control (no probiotic)	0.00 ± 1.11	—
*L. reuteri *NCIMB 11951	13.13 ± 3.61	0.33 ± 0.09^e^
*L. reuteri *NCIMB 701359	14.63 ± 1.14	0.19 ± 0.02^e^
*L. reuteri *NCIMB 702655	19.55 ± 1.45	0.96 ± 0.07^d,e^
*L. reuteri *NCIMB 701089	20.87 ± 2.44	0.99 ± 0.12^d,e^
*L. reuteri *NCIMB 702656	38.99 ± 4.87	2.09 ± 0.26^d^
*L. fermentum *NCIMB 5221	23.55 ± 2.05	10.45 ± 0.91^b^
*L. fermentum *NCIMB 8829	36.06 ± 1.49	1.16 ± 0.05^d,e^
*L. fermentum *NCIMB 2797	28.48 ± 0.68	3.81 ± 0.09^c^
*L. rhamnosus *ATCC 53103 GG	29.98 ± 4.03	0.52 ± 0.07^d,e^
*L. acidophilus *ATCC 314	31.51 ± 1.39	1.79 ± 0.08^d,e^
*L. plantarum *ATCC 14917	28.3 ± 1.03	15.18 ± 0.55^a^

Data is expressed as mean ± SEM, *n* = 3. Tukey's homogeneous subsets generated from pairwise comparisons are represented as a, b, c, d, and e, with “a” representing the most significant subset from control.

**Table 3:** Percent cholesterol assimilation by *Lactobacillus *strains in simulated intestinal fluid containing 100 *μ*g/mL of cholesterol-PEG 600 for 24 h and the amount of cholesterol assimilation expected in a probiotic dose containing 10^10^ cells.

Probiotic strain	Cholesterol assimilated (%)	Cholesterol assimilated (mg/10^10^ cfu)
Control (no probiotic)	0.00 ± 1.87	—
*L. reuteri *NCIMB 11951	63.24 ± 1.29	25.02 ± 0.51^c^
*L. reuteri *NCIMB 701359	50.77 ± 1.67	139.63 ± 4.59^c^
*L. reuteri *NCIMB 702655	52.69 ± 2.42	803.62 ± 36.85^b^
*L. reuteri *NCIMB 701089	67.20 ± 1.89	2254.70 ± 63.33^a^
*L. reuteri *NCIMB 702656	51.79 ± 1.52	20.94 ± 0.61^c^
*L. fermentum *NCIMB 5221	11.51 ± 1.44	137.94 ± 17.29^c^
*L. fermentum *NCIMB 8829	37.19 ± 4.99	43.62 ± 5.85^c^
*L*. *fermentum *NCIMB 2797	40.84 ± 1.90	236.24 ± 10.98^c^
*L. rhamnosus *ATCC 53103 GG	−0.76 ± 1.09	−14.19 ± 20.39^c^
*L. acidophilus *ATCC 314	73.67 ± 3.43	247.17 ± 11.51^c^
*L. plantarum *ATCC 14917	20.54 ± 3.85	1148.50 ± 215.32^b^

Data is expressed as mean ± SEM, *n* = 3. Tukey's homogeneous subsets generated from pairwise comparisons are represented as a, b, and c, with “a” representing the most significant subset from control.
